# Phase 2 trial with imeglimin in patients with Type 2 diabetes indicates effects on insulin secretion and sensitivity

**DOI:** 10.1002/edm2.371

**Published:** 2022-10-14

**Authors:** Pierre Theurey, Carole Thang, Valdis Pirags, Andrea Mari, Giovanni Pacini, Sébastien Bolze, Sophie Hallakou‐Bozec, Pascale Fouqueray

**Affiliations:** ^1^ Poxel SA Lyon France; ^2^ University of Latvia Riga Latvia; ^3^ Institute of Neuroscience National Research Council Padova Italy; ^4^ Independent Researcher Padova Italy

**Keywords:** diabetes, insulin resistance, mitochondria

## Abstract

**Introduction:**

The aim of the present study was to evaluate the effect of 18‐week monotherapy with imeglimin on glucose tolerance and on insulin secretion/sensitivity in type 2 diabetic (T2D) patients.

**Methods:**

The study was an 18‐week, double‐blind clinical trial in T2D subjects previously treated with stable metformin therapy and washed out for 4 weeks. Subjects were randomized 1:1 to receive a 1500 mg bid of imeglimin or placebo. The primary endpoint was the effect of imeglimin vs placebo on changes from baseline to week 18 in glucose tolerance (glucose area under the curve [AUC]) during a 3 h‐glucose tolerance test [OGTT]). Secondary endpoints included glycaemic control and calculated indices of insulin secretion and sensitivity.

**Results:**

A total of 59 subjects were randomized, 30 receiving imeglimin and 29 receiving placebo. The study met its primary endpoint. Least squares (LS) mean difference between treatment groups (imeglimin ‐ placebo) for AUC glucose from baseline to week 18 was −429.6 mmol/L·min (*p* = .001). Two‐hour post‐dose fasting plasma glucose was significantly decreased with LS mean differences of −1.22 mmol/L (*p* = .022) and HbA1c was improved with LS mean differences of −0.62% (*p* = .013). The AUC_0‐180min_ ratio C‐peptide/glucose [LS mean differences of 0.041 nmol/mmol (*p* < .001)] and insulinogenic index were significantly increased by imeglimin treatment. The increase in insulin secretion was associated with an increase in beta‐cell glucose sensitivity. Additionally, the insulin sensitivity indices derived from the OGTT Stumvoll (*p* = .001) and Matsuda (not significant) were improved in the imeglimin group vs placebo. Imeglimin was well tolerated with 26.7% of subjects presenting at least one treatment‐emergent adverse event versus 58.6% of subjects in the placebo group.

**Conclusions:**

Results are consistent with a mode of action involving insulin secretion as well as improved insulin sensitivity and further support the potential for imeglimin to improve healthcare in T2D patients.

## Graphical abstract

This article describes the results of a 18‐week double‐blind clinical trial, assessing the effects of imeglimin on insulin secretion and sensitivity using an oral glucose tolerance test in Caucasian Type 2 Diabetic patients. It reports for the first time the effect of imeglimin on dynamic insulin sensitivity and on insulin secretion using a sophisticated mathematical approach.

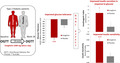

## 1 INTRODUCTION

Despite major advances in biomedical research and a robust drug portfolio, the type 2 diabetes (T2D) “pandemic” continues to grow at an alarming rate and results in extensive morbidity and the death of millions of patients every year,^1^ underscoring a persistent need for further innovation in therapeutic strategies.

Mitochondria are central and crucial organelles controlling energy and metabolic homeostasis. Extensive evidence has pointed toward impairment of mitochondrial content, function and structure and subsequent increases in oxidative stress as key players in the pathophysiology of metabolic diseases including T2D.^2^, ^3^ Accordingly, numerous antidiabetic agents directly or indirectly improve mitochondrial function.^4^

Imeglimin is the first in a new class of tetrahydrotriazine‐based family of oral antidiabetics: the glimins (World Health Organization: ATC 5th level ‐ A10BX15 imeglimin), targeting mitochondrial dysfunction. In this context, imeglimin has demonstrated a positive effect on mitochondrial function in diabetic mice through mild and competitive inhibition of complex I and rescue of complex III activity/protein content, and inhibition of reactive oxygen species production.^5^, ^6^, ^7^

Several lines of evidence have shown the beneficial effects of imeglimin on pancreatic β‐cell mass and function. Preclinical studies showed that imeglimin improved hyperglycaemia and enhanced glucose‐stimulated insulin secretion (GSIS) using multiple rodent models of T2D (isolated islets and intact animals), including models characterized by a primary defect in β‐cell mass and function.^6^, ^8^, ^9^, ^10^, ^11^ Importantly, investigations in isolated islets from T2D rodents have shown that imeglimin acts via a novel mechanism of action involving activation of the NAD^+^ salvage pathway, by inducing the nicotinamide phosphoribosyltransferase (NAMPT), increasing the NAD^+^ pool as well as ATP levels in response to glucose in parallel.^8^ The ADP ribosyl cyclase/cADPR hydrolase (CD38) further converts NAD^+^ in cyclic ADP ribose (cADPR),^8^ activating the transient receptor potential melastatin 2 (TRPM2) channel, which increases the nonselective cation channels (NSCC) current for insulinotropic action.^12^ Accordingly, direct clinical evidence of an effect to enhance β‐cell function was unveiled with a hyperglycaemic clamp performed in T2D patients, in which substantial improvements in GSIS occurred following a 1‐week treatment period.^13^ Other clinical results support the effect of imeglimin on insulin secretion, such as decreases in the pro‐insulin/C‐peptide ratio and increase of HOMA‐β (index of β‐cell insulin secretory function under fasted conditions) in phase 2 and phase 3 trials.^14^, ^15^, ^16^ Importantly, imeglimin also demonstrated protection of β‐cell mass in rodent T2D in vivo model systems and β‐cell protection in the context of cytotoxic insults applied to rodent and human islets in vitro ^10^, ^11^, ^17^.

Several clues point toward an effect of imeglimin on insulin sensitivity in preclinical models as well. In a mouse model of diet‐induced T2D, imeglimin enhanced the actions of intraperitoneal injection of insulin on glycaemia, improved insulin signalling in muscle and liver, and reduced hepatic steatosis.^6^ Additionally, imeglimin enhanced glucose uptake in muscle in vivo ^11^. Importantly, a substantial improvement in whole‐body insulin sensitivity and hepatic glucose production was observed during a hyperinsulinaemic clamp in STZ‐diabetic rats.^18^

Beyond its action on insulin GSIS and insulin sensitivity, imeglimin exerted positive effects on heart and kidney function and structure. In rat models mimicking human metabolic syndrome, imeglimin improved cardiac diastolic and vascular dysfunction, as well as myocardial and kidney structure.^19^ In mouse models of heart failure with preserved ejection fraction (HFpEF), imeglimin showed positive effects on cardiac abnormalities.^20^ The underlying molecular mechanisms involved were reduction of oxidative stress, improvement of nitric oxide homeostasis and rescue of disrupted unfolded protein response.^19^, ^20^

The current portfolio of available oral drugs to treat T2D is comprised of multiple drug classes targeting the different elements of the disease pathogenesis by reducing hepatic gluconeogenesis, indirectly and directly promoting insulin secretion, promoting renal glucose excretion, and improving peripheral insulin resistance.^21^ In this landscape, imeglimin stands out by exhibiting a dual effect on both glucose‐stimulated insulin secretion highlighted in preclinical^11^ and in T2D patients,^13^ and insulin sensitivity in muscle and liver in preclinical models,^18^ associated with a preclinical cardiorenal benefit.^19^

The following evidence from phase 1 and phase 2 clinical trials, the efficacy and favourable safety profile of imeglimin was confirmed during the pivotal phases 3 program TIMES (Trial for Imeglimin Efficacy and Safety) in Japan: as monotherapy ‐ TIMES 1,^15^ as combination therapy with other antidiabetics ‐ TIMES 2,^22^ and as an add‐on to insulin monotherapy ‐ TIMES 3.^23^ Imeglimin was approved in June 2021 in Japan, constituting a new therapeutic option as first‐line therapy or as a complementary treatment for patients already treated with all oral or injectable antidiabetic therapies (including insulin).^24^ It constitutes as well the first new oral T2D therapeutic class since the advent of SGLT2 inhibitors more than a decade earlier.

In the present study, we describe the results of an 18 weeks double‐blind (DB) clinical trial, assessing the effects of a 1500 mg bid of imeglimin on fasting and postprandial glucose control, investigating insulin secretion and sensitivity using an OGTT in Caucasian T2D patients that were on metformin monotherapy and washed out for 4 weeks before the start of imeglimin treatment.

## 2 METHODS

### 2.1 Study design and participants

This was a phase 2, multi‐centre, double‐blind, placebo‐controlled, randomized, parallel groups study (EudraCT number: 2013‐001539‐35) conducted at 9 centres in 3 countries (4 centres in Hungary, 2 centres in Latvia and 3 centres in Romania). This study was approved by the Independent Ethics Committee of each country according to national regulations and was conducted in accordance with the ethical principles of the Declaration of Helsinki, as well as with the International Conference on Harmonization (ICH) Note for Guidance on Good Clinical Practice (GCP; ICH Topic E6, 1996) and all applicable local regulatory requirements. Subjects had given written informed consent before any study‐related activities.

The study was performed on 18–75 years old subjects with T2D, treated with metformin monotherapy at a minimum dose of 1500 mg/day and stable for the previous 12 weeks prior to screening, with HbA1c between 7.2% and 9.5% and glomerular filtration rate (GFR) as estimated by the Modified Diet in Renal Disease (MDRD) formula ≥60 ml/min/1.73 m^2^. Key exclusion criteria included treatment with any other anti‐diabetic treatment than metformin within 12 weeks before the screening, an acute cardiovascular event within 6 months before screening and retinopathy of severity above mild non‐proliferative diabetic retinopathy.

### 2.2 Procedures

**FIGURE 1:**
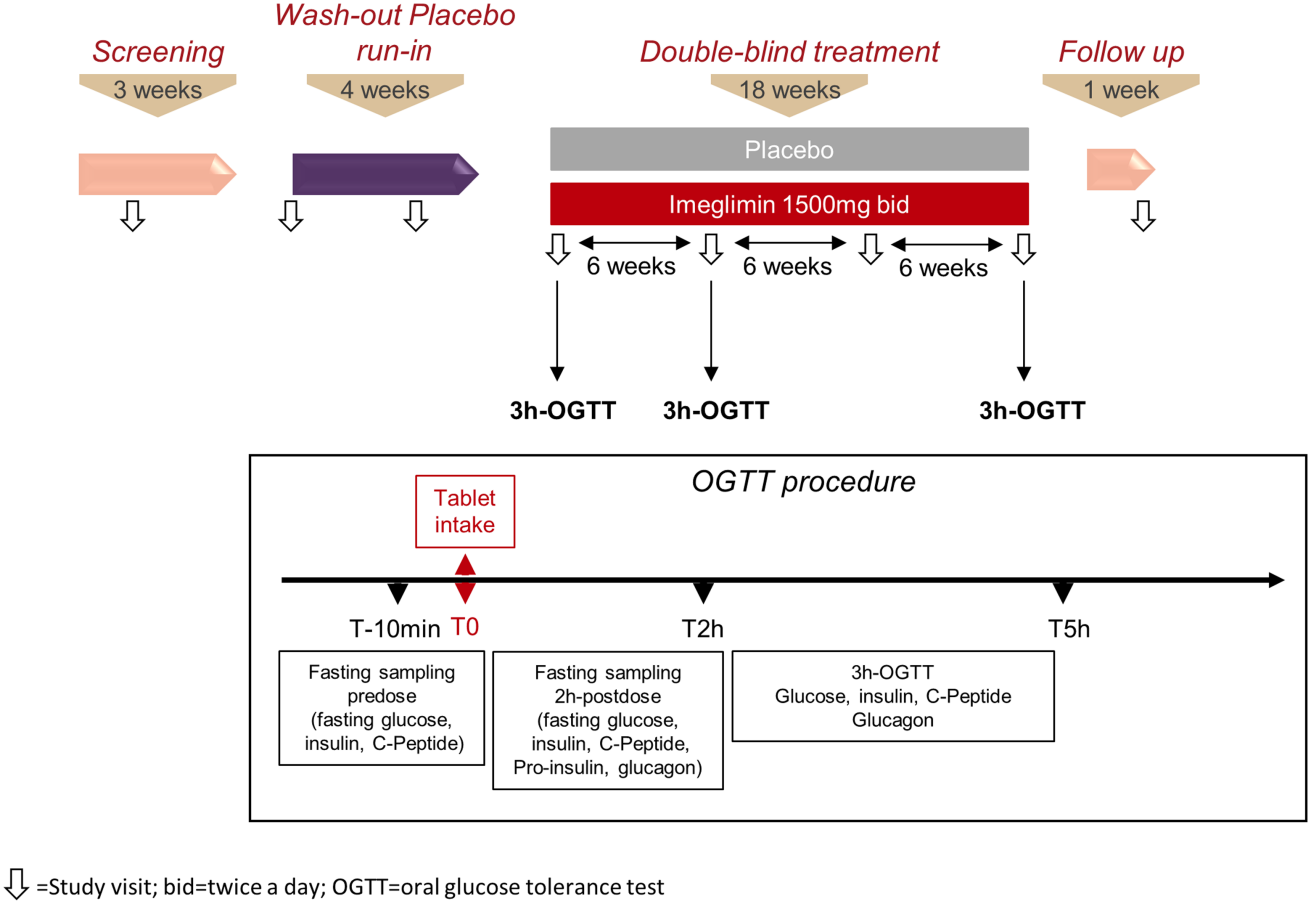
Study design

Study design is represented in Figure 1.

Following the screening period, subjects underwent a 4 weeks wash‐out period (single‐blind, 3 tablets of placebo bid). The subjects were then randomized in a 1:1 ratio to receive either imeglimin (1500 mg bid) or placebo in a DB manner for 18 weeks, followed by a 1 weeks follow‐up period after the last intake of the double‐blind treatment. Appropriate anti‐diabetic treatment was then initiated at the discretion of the investigator.

Three 3 h oral glucose tolerance tests (OGTTs) were performed in the morning and in the fasted state 2 h after dosing with study medication. OGTTs were performed during the baseline visit on day one of the DB treatment period, after 6 weeks, and after 18 weeks at the end of treatment.

At baseline, 6 weeks and 18 weeks, fasting plasma glucose (FPG), insulin, C‐peptide and glucagon were measured 5 min before glucose load, and at 15, 30, 60, 90, 120 and 180 min post‐load for glucose, insulin and C‐peptide, and at 30, 60, 120 and 180 min post‐load for glucagon.

If glucose monitoring showed deterioration of glycaemic control during the study, and plasma glucose and HbA1c values were above the threshold values (following FDA recommendations), rescue therapy with a commonly used anti‐diabetic agent (including insulin) was allowed and the subject was withdrawn from the study.

During the placebo wash‐out period, an FPG greater than 270 mg/dl (15 mmol/L) was considered as a threshold to initiate rescue therapy. During the DB treatment period, the following were considered as thresholds to initiate rescue therapy:

• FPG greater than 270 mg/dl (15 mmol/L) from baseline to week 6

• FPG greater than 240 mg/dl (13.3 mmol/L) from week 6 to week 12

• FPG greater than 200 mg/dl (11.1 mmol/L) or HbA1c greater than 9% from week 12 to the end of treatment

### 2.3 Outcomes

The primary objective of this study was to assess the effect of imeglimin versus placebo on glucose tolerance ‐ plasma glucose area under the curve (AUC) ‐ during a 3 h OGTT, following 18 weeks of treatment. AUC was calculated using the linear trapezoidal rule.

Secondary objectives of this study included assessment of the effects of the 18 weeks imeglimin treatment versus placebo on additional glycaemic parameters: HbA1c, FPG, insulin, C‐peptide and glucagon, fasting or during the 3 h OGTTs.

From the above OGTT measures, the β‐cell function was evaluated using the ratio AUC_0‐180min_ C‐peptide/AUC_0‐180min_ glucose and the insulinogenic index,^25^ calculated as the ratio of insulin to plasma glucose 0–30 min after a glucose load.

Additional post hoc efficacy analyses were performed to obtain further assessments of β‐cell function. AUC for total insulin secretion rate (ISR) was calculated by deconvolution of C‐peptide peripheral concentrations (through the Van Cauter approach^26^). A mathematical model describing the relationship between ISR and plasma glucose concentration measured during the OGTT was applied. This model has been widely described and exploited to characterize β‐cell activity.^27^, ^28^, ^29^ From this model, the β‐cell dose–response curve (i.e. the dose–response function of ISR vs plasma glucose concentration) was determined and the following parameters/indices were calculated:

- The insulin response to the rate of change in glucose concentration is called rate sensitivity, which is a surrogate index of first‐phase insulin secretion.
- Insulin secretion at a reference glucose concentration of 10 mmol/L (representing an average fasting glucose value in the whole study group), calculated from the β‐cell dose–response curve.
- Glucose sensitivity, i.e. the slope of the β‐cell dose–response curve, which represents an index on how well the β‐cell responds to the glucose stimulation.

Insulin sensitivity was evaluated both fasting using the QUICKI (predose) and during the OGTT using the oral glucose insulin sensitivity (OGIS ‐ 2 h equation), Stumvoll ISI_est_ and Matsuda indices ‐ calculated as previously described.^30^

The safety and tolerability profile of imeglimin and placebo were assessed through the recording, reporting and analysis of baseline medical conditions, adverse events (AEs), physical examination findings, BMI, weight and waist circumference, vital signs, ECG and laboratory tests. A comprehensive assessment of AEs experienced by the subject was performed throughout the course of the study, from the time of informed consent until the end of the post‐treatment follow‐up period, defined as the end‐of‐study visit. Each AE was classified using the Medical Dictionary for Regulatory Activities (version 17.1 ‐ MedDRA®). Subjects were provided with a blood glucose self‐monitoring device and appropriate material to check their blood glucose on a regular basis as well as a diary to report their values and any symptoms.

### 2.4 Statistics

A sample size of 25 subjects per treatment group was based on previous experience with imeglimin in exploratory monotherapy studies with similar sample sizes which had shown significant changes from baseline in glucose AUC during the OGTT. Assuming a dropout rate of 20%, a total of 30 subjects per treatment group were to be randomized in the study to ensure that at least 25 subjects per treatment group complete the study.

Safety and tolerability were assessed using the safety population, including all subjects who received at least one double‐blind dose of imeglimin or placebo. Efficacy analyses were primarily performed on the intent‐to‐treat (ITT) population, including all subjects who received at least one double‐blind dose of imeglimin or placebo and provided a baseline and at least one post‐baseline assessment of either the primary or secondary efficacy parameters. When applicable, a last observation carried forward (LOCF) was implemented in the ITT population analysis to replace missing parameter values for all those subjects who did not present a data value at week 18. The LOCF imputation was defined as follows: the last (looking at date/time) non‐missing postbaseline scheduled visit value during the double‐blind treatment period was carried forward and used for all subsequent visits where the value was missing.

A Shapiro–Wilk test was performed to test normality. Data that did not follow the normal distribution were transformed on the natural logarithmic scale for analysis and estimates of differences between treatment groups were back‐transformed to provide relative effects. Changes from baseline to week 18 were assessed with an analysis of covariance (ANCOVA) model. Two‐sided statistical tests were performed to compare these changes between the two groups, with a nominal significance level of 0.05. The baseline adjusted mean difference between treatments was presented along with the corresponding standard error of mean and 95% CI.

## 3 RESULTS

### 3.1 Patient disposition and baseline characteristics

**FIGURE 2:**
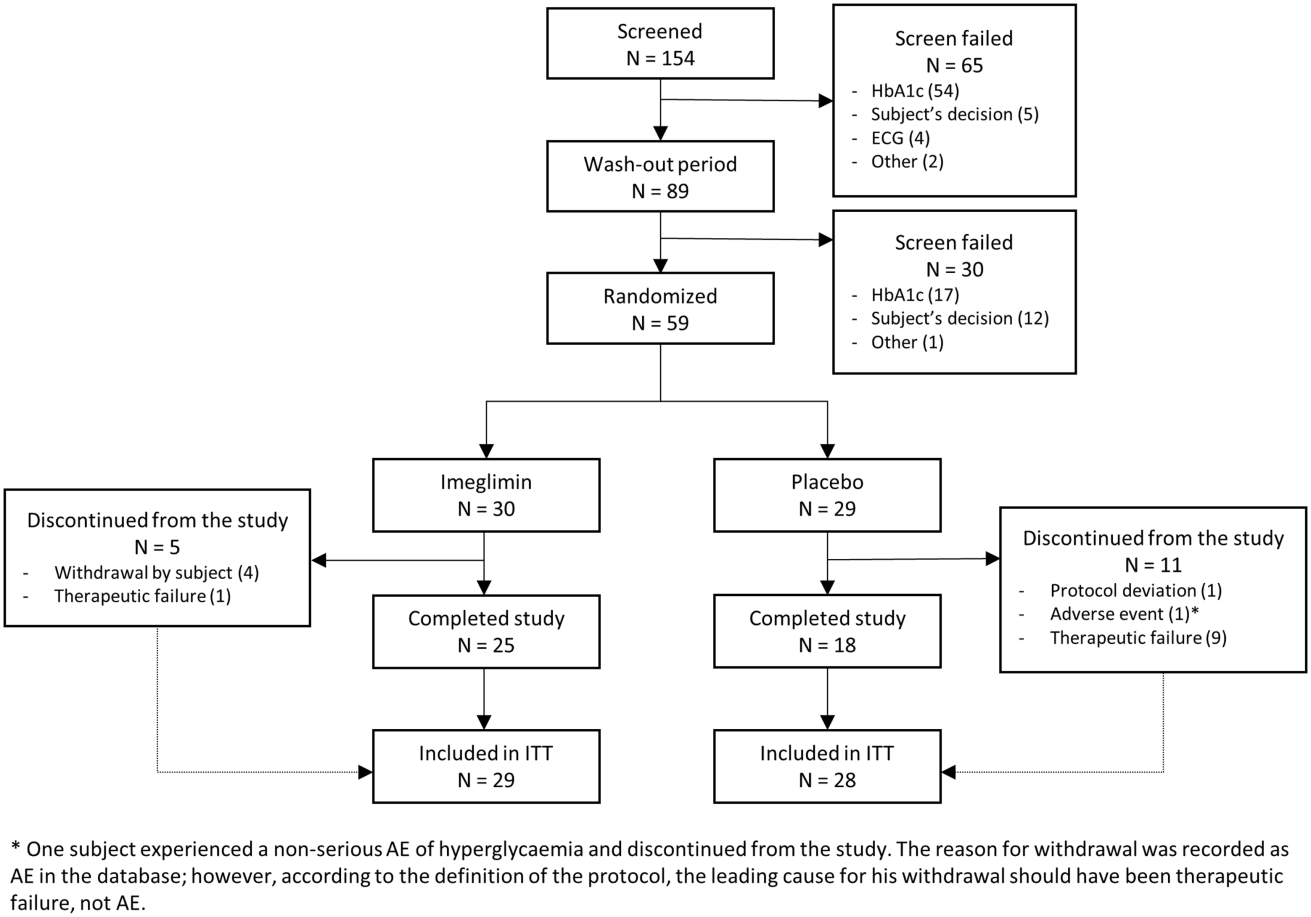
Patient disposition

The clinical trial was initiated on 12 August 2013 and completed on 23 October 2014. Of 154 subjects screened for inclusion, 59 subjects were randomly assigned to treatment (Figure 2). Of these, 43 (72.9%) completed the study. Among the 59 randomized subjects, 57 received at least 1 dose of DB study medication (imeglimin or placebo), provided a baseline and at least 1 postbaseline assessment of either the primary or key secondary efficacy parameters, and were included in the ITT population analysis (Figure 2). A total of 16 (27.1%) subjects discontinued from the study, including 5 (16.7%) subjects in the imeglimin group and 11 (37.9%) subjects in the placebo group. The most frequent reasons for premature treatment discontinuation were therapeutic failure requiring an urgent adjustment in the glucose‐lowering treatment and withdrawal by the subject (Figure 2). Baseline characteristics were similar between treatment groups (Table 1).

**TABLE 1:** Demographic and baseline characteristics

Characteristic	Imeglimin (*N* = 30)	Placebo (*N* = 29)	Overall (*N* = 59)
Age (years)	58.4 (8.0)	54.3 (8.9)	56.4 (8.6)
Gender			
Male	12 (40.0%)	16 (55.2%)	28 (47.5%)
Female	18 (60.0%)	13 (44.8%)	31 (52.5%)
Ethnic Origin: Not Hispanic or Latino	30 (100%)	29 (100%)	59 (100%)
Race: White	30 (100%)	29 (100%)	59 (100%)
Weight (kg)	90.83 (16.10)	93.21 (17.00)	92.00 (16.45)
BMI (kg/m^2^)	32.83 (4.95)	32.91 (4.26)	32.87 (4.58)
eGFR (MDRD, ml/min/1.73 m^2^)	97.5 (19.0)	99.7 (20.1)	98.6 (19.4)
Duration of diabetes (years)	5.9 (4.5)	5.0 (2.8)	5.4 (3.8)
Metformin daily dose (mg)	2040.0 (338.7)	2037.9 (364.9)	2039.0 (348.8)
HbA1c (%)			
At screening (before washout)	7.74 (0.47)	7.86 (0.60)	7.80 (0.54)
At randomization (baseline)	8.12 (0.56)	8.14 (0.61)	8.13 (0.58)
Fasting plasma glucose (mmol/L)	11.33 (2.53)	10.25 (1.92)	10.80 (2.99)

*Note*: Data are reported as mean (SD) or *n* (%).

Abbreviations: BMI, body mass index; eGFR, estimated glomerular filtration rate; For HbA1c and FPG, *n*, 29 and 28, respectively (ITT pop); HbA1c, glycated haemoglobin; MDRD, modified diet in renal disease; SD, standard deviation.

### 3.2 Glucose tolerance during OGTT

**FIGURE 3:**
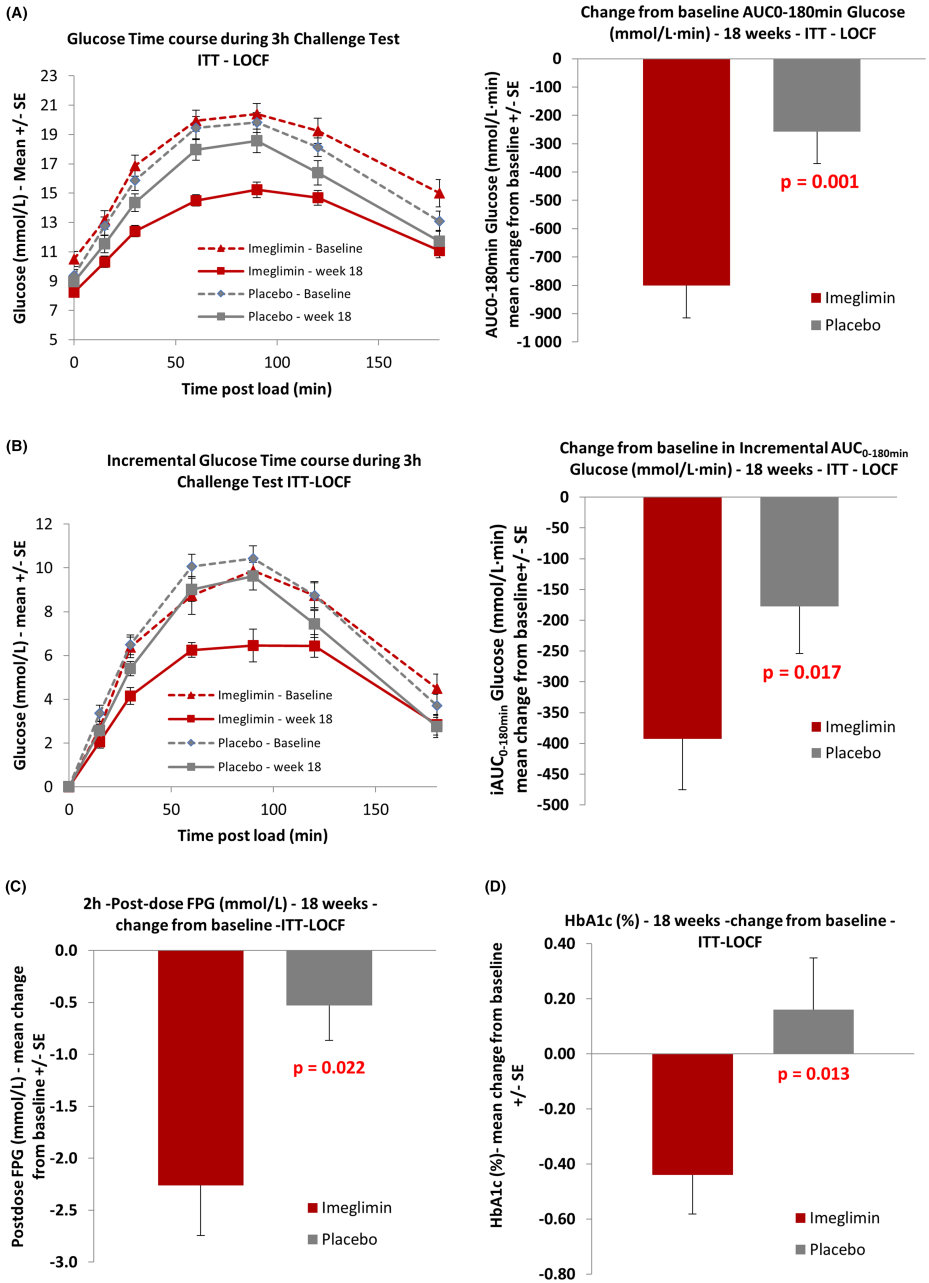
Effect of imeglimin on glucose tolerance and glycaemic parameters

The study met its primary endpoint. Least squares (LS) mean change for AUC glucose during the OGTT from baseline to week 18 was −720.7 and −291.0 mmol/L·min in the imeglimin and placebo groups, respectively. LS mean difference between treatment groups (imeglimin ‐ placebo) was −429.6 (95% CI: −678.0, −181.3) mmol/L·min, and was statistically significant (*p* = .001, Figure 3A, Table 2). Incremental AUC was reduced as well in the imeglimin group compared to placebo. LS mean change from baseline to week 18 was −369.6 and −149.3 mmol/L·min in the imeglimin and placebo groups, respectively. LS mean difference between treatment groups was −220.3 (95% CI: −399.6, −41.0) mmol/L·min, and was statistically significant (*p* = .017, Figure 3B, Table 2).

**TABLE 2:** Effects of imeglimin versus placebo on primary and secondary efficacy end points

	Imeglimin	Placebo
180‐min AUC Glucose (mmol/L·min), *n*	29	27
Baseline, mean (SD)	3181.0 (679.0)	3005.6 (552.0)
Change from baseline, LSM	−720.7	−291.0
Difference vs. placebo, LSM (95% CI)	−429.6 (−678.0, −181.3)	
	*p* = .001	
180‐min iAUC Glucose (mmol/L·min), *n*	29	27
Baseline, mean (SD)	1291.6 (381.2)	1315.7 (379.2)
Change from baseline, LSM	−369.6	−149.3
Difference vs. placebo, LSM (95% CI)	−220.3 (−399.6, −41.0)	
	*p* = .017	
HbA1c (%), *n*	29	28
Baseline, mean (SD)	8.12 (0.56)	8.14 (0.61)
Change from baseline, LSM	−0.46	0.17
Difference vs. placebo, LSM (95% CI)	−0.62 (−1.11, −0.14)	
	*p* = .013	
Pre‐dose Fasting Plasma Glucose (mmol/L), *n*	29	28
Baseline, mean (SD)	11.33 (2.53)	10.25 (1.92)
Change from baseline, LSM	−1.59	−0.69
Difference vs. placebo, LSM (95% CI)	−0.91 (−1.97, −0.15)	
	*p* = .092	
Post‐dose Fasting Plasma Glucose (mmol/L), *n*	29	26
Baseline, mean (SD)	10.51 (2.74)	9.45 (2.02)
Change from baseline, LSM	−2.01	−0.80
Difference vs. placebo, LSM (95% CI)	−1.22 (−2.25, −0.18)	
	*p* = .022	
Pre‐dose Fasting Insulin (pmol/L), *n*	29	27
Baseline, mean (SD)	11.83 (6.80)	19.16 (17.32)
Change from baseline, LSM	−2.51	−1.84
Difference vs. placebo, LSM (95% CI)	−0.67 (−3.58, 2.24)	
	*p* = .646	
Post‐dose Fasting Glucagon (pmol/L), *n*	19	18
Baseline, mean (SD)	95.6 (30.79)	106.8 (37.92)
Change from baseline, LSM	−2.0	−4.5
Difference vs. placebo, LSM (95% CI)	2.5 (−12.3, 17.3)	
	*p* = .730	
180‐min AUC Insulin (pmol/L·min), *n*	29	26
Baseline, mean (SD)	5427.3 (2555.0)	6888.2 (4262.7)
Change from baseline, LSM	686.6	307.6
Difference vs. placebo, LSM (95% CI)	379.1 (−1203.4, 1961.5)	
	*p* = .633	
180‐min AUC C‐peptide (nmol/L·min), *n*	28	27
Baseline, mean (SD)	369.0 (121.7)	379.0 (142.0)
Change from baseline, LSM	44.5	−0.0
Difference vs. placebo, LSM (95% CI)	44.5 (−0.4, 89.5)	
	*p* = .052	
Stumvoll index, *n*	29	27
Baseline, mean (SD)	0.0439 (0.0159)	0.0441 (0.0160)
Change from baseline, LSM	0.0182	0.0049
Difference vs. placebo, LSM (95% CI)	0.0133 (0.0058, 0.0208)	
	*p* = .001	
Matsuda index, *n*	28	23
Baseline, mean (SD)	2.84 (2.04)	2.82 (5.01)
Change from baseline, LSM	1.54	−0.02
Difference vs. placebo, LSM (95% CI)	1.55 (−1.06, 4.17)	
	*p* = .237	
OGIS index (2‐h equation ‐ ml/min/m^2^), *n*	27	23
Baseline, mean (SD)	267.42 (49.121)	281.62 (45.527)
Change from baseline, LSM	25.96	18.89
Difference vs. placebo, LSM (95% CI)	7.08 (−21.16, 35.32)	
	*p* = .616	
QUICKI, *n*	29	27
Baseline, mean (SD)	0.218 (0.034)	0.208 (0.037)
Change from baseline, LSM	0.014	0.002
Difference vs. placebo, LSM (95% CI)	0.012 (−0.008, −0.031)	
	*p* = .244	
Insulinogenic index (pmol/mmol), *n*	28	23
Baseline, mean (SD)	2.39 (1.96)	2.52 (2.35)
Change from baseline, LSM	2.55	0.76
Difference vs. placebo, LSM (95% CI)	1.79 (0.24, 3.34)	
	*p* = .025	
AUC0‐180 min C‐peptide / AUC0‐180 min glucose (nmol/mmol), *n*	28	27
Baseline, mean (SD)	0.122 (0.048)	0.137 (0.083)
Change from baseline, LSM	0.054	0.012
Difference vs. placebo, LSM (95% CI)	0.041 (0.023, 0.060)	
	*p* < .001	
Rate sensitivity (pmol/m^2^/mmol/L), *n*	29	27
Baseline, mean (SD)	226.64 (209.62)	207.35 (210.37)
Change from baseline, LSM	211.61	28.36
Difference vs. placebo, LSM (95% CI)	183.25 (−1.17, 367.67)	
	*p* = .051	
Insulin Secretion at 10 mmol/L Glucose from the Dose–Response (pmol/min/m^2^), *n*	29	27
Baseline, mean (SD)	132.88 (70.24)	178.10 (140.89)
Change from baseline, LSM	65.79	11.31
Difference vs. placebo, LSM (95% CI)	54.48 (14.68, 94.28)	
	*p* = .008	
Glucose sensitivity (pmol/min/m^2^/mmol/L), *n*	29	27
Baseline, mean (SD)	20.41 (9.45)	24.13 (25.38)
Change from baseline, LSM	14.54	2.29
Difference vs. placebo, LSM (95% CI)	12.25 (4.20, 20.30)	
	*p* = .004	

Abbreviations: AUC, area under the curve; CI, confidence interval; HbA1c, glycated haemoglobin; HOMA‐IR, homeostatic model assessment for insulin resistance; iAUC, incremental area under the curve; LSM, least square mean; SD, standard deviation.

### 3.3 Other glycaemic parameters

During the 18 weeks treatment, imeglimin showed a significantly larger reduction in HbA1c and FPG 2 hours post‐dose compared to placebo (Table 2, Figure 3C and D). The decrease in pre‐dose FPG did not reach statistical significance (*p* = .092, Table 2).

### 3.4 Insulin secretion in response to glucose

**FIGURE 4:**
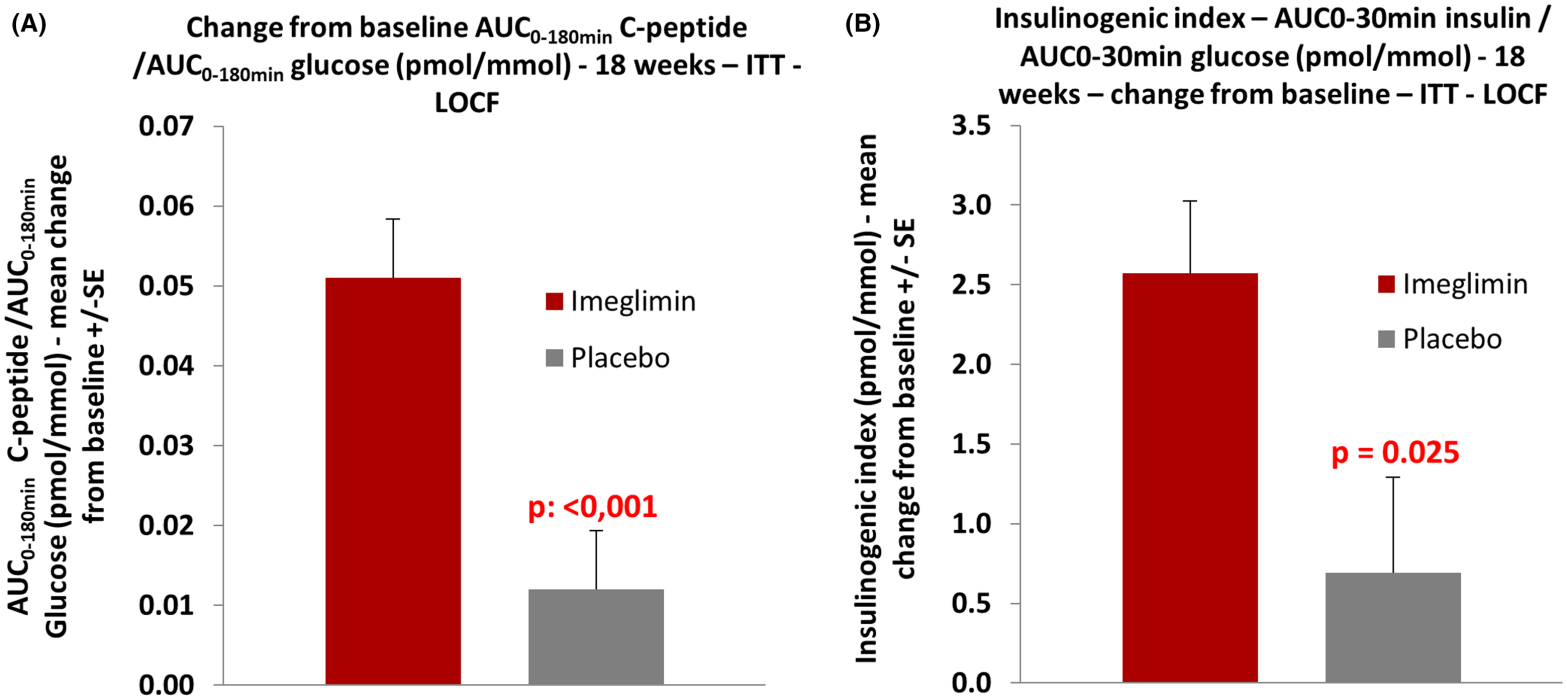
Effect of imeglimin on insulin secretion in response to glucose

To elucidate the mechanisms by which imeglimin exerted positive effects on glycaemic control, we calculated multiple parameters to assess its action on insulin secretion and β‐cell function. Significant improvement was observed. LS mean change from baseline to week 18 for the ratio AUC_0‐180min_ C‐peptide/AUC_0‐180min_ glucose was 0.054 and 0.012 nmol/mmol in the imeglimin and placebo groups, respectively, showing a marked improvement of the dynamic β‐cell function. LS mean difference between treatment groups was 0.041 (95% CI: 0.023, 0.060) nmol/mmol, which was statistically significant (*p* < .001, Figure 4A, Table 2). Early insulin response to glucose during the OGTT (0–30 minutes) was stimulated by imeglimin: LS mean change from baseline to week 18 for the insulinogenic index was 2.55 and 0.76 pmol/mmol in the imeglimin and placebo groups, respectively. LS mean difference between treatment groups (imeglimin ‐ placebo) was 1.79 (95% CI: 0.24, 3.34) pmol/mmol, which reached the statistical significance level (*p* = .025, Figure 4B, Table 2).

**FIGURE 5:**
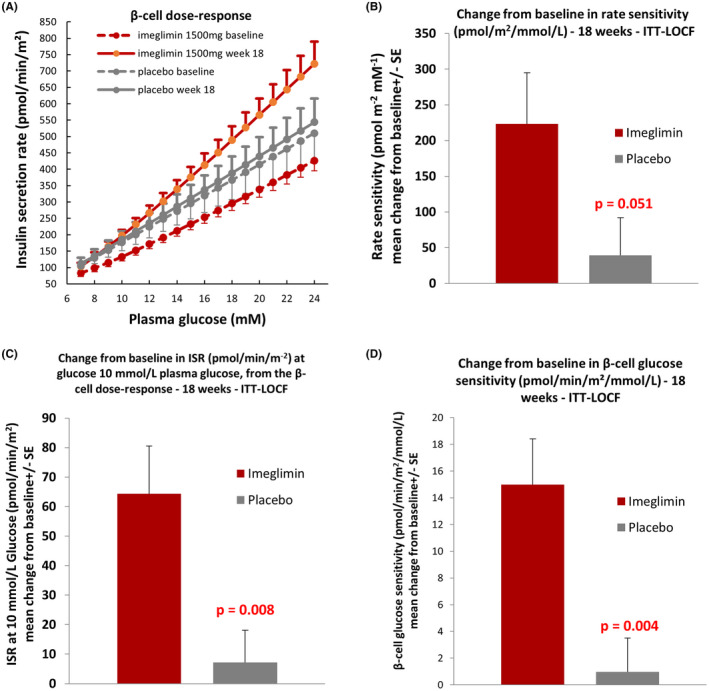
Effect of imeglimin on the β‐cell function

We performed additional post hoc efficacy analyses to estimate β‐cell function with some more sophisticated techniques. In line with the above results, the beneficial effects of imeglimin were confirmed. LS mean change from baseline to week 18 in rate sensitivity, a marker of first‐phase insulin secretion, was 211.61 and 28.36 pmol/m^2^/mmol/L in the imeglimin and placebo groups, respectively. LS mean difference between treatment groups was 183.25 (95% CI: −1.17, 367.67) pmol/m^2^/mmol/L, which was very close to statistical significance (*p* = .051, Figure 5B, Table 2). Moreover, imeglimin improved the ISR at 10 mmol/L plasma glucose concentration, calculated from the dose–response function of ISR vs plasma glucose concentration (β‐cell dose–response curves plotted in Figure 5A). LS mean change from baseline to week 18 was 65.79 and 11.31 pmol/min/m^2^ in the imeglimin and placebo groups, respectively. LS mean difference between treatment groups was 54.48 (95% CI: 14.68, 94.28) pmol/min/m^2^, which was statistically significant (*p* = .008, Figure 5C, Table 2). Imeglimin improved β‐cell glucose sensitivity (slope of the β‐cell dose–response ‐ ISR vs plasma glucose curves plotted in Figure 5A). LS mean change from baseline to week 18 was 14.54 and 2.29 pmol/min/m^2^/mmol/L in the imeglimin and placebo groups, respectively. LS mean difference between treatment groups was 12.25 (95% CI: 4.20, 20.30) pmol/min/m^2^/mmol/L, which was statistically significant (*p* = .004, Figure 5D, Table 2). Thus, it was clearly confirmed that imeglimin markedly improved all the aspects of β‐cell function.

Of note, imeglimin treatment had no apparent effect on basal or OGTT‐associated insulin clearance (not shown), highlighting that the effect of the drug on insulinemia is solely related to insulin secretion. Imeglimin treatment also had no effects on fasting insulin levels or on fasting and OGTT stimulated glucagon levels (Table 2).

### 3.5 Other parameters during OGTT

No statistically significant change in insulin levels was observed during the OGTT following 18‐week imeglimin treatment, compared to placebo. However, LS mean change from baseline to week 18 for AUC insulin was 686.6 and 307.6 pmol/L·min in the imeglimin and placebo groups, respectively, and LS mean difference between treatment groups was 379.1 (95% CI: −1203.4, 1961.5) pmol/L·min. The difference was not significant due to high variability (*p* = .633, Figure S1A, Table 2). For AUC C‐peptide, LS mean change from baseline to week 18 was 44.5 and 0 mmol/L·min in the imeglimin and placebo groups, respectively. LS mean difference between treatment groups was 44.5 (95% CI: −0.4, 89.5) mmol/L·min. The difference was very close to statistical significance (*p* = .052, Figure S1A, Table 2).

### 3.6 Insulin sensitivity indices

**FIGURE 6:**
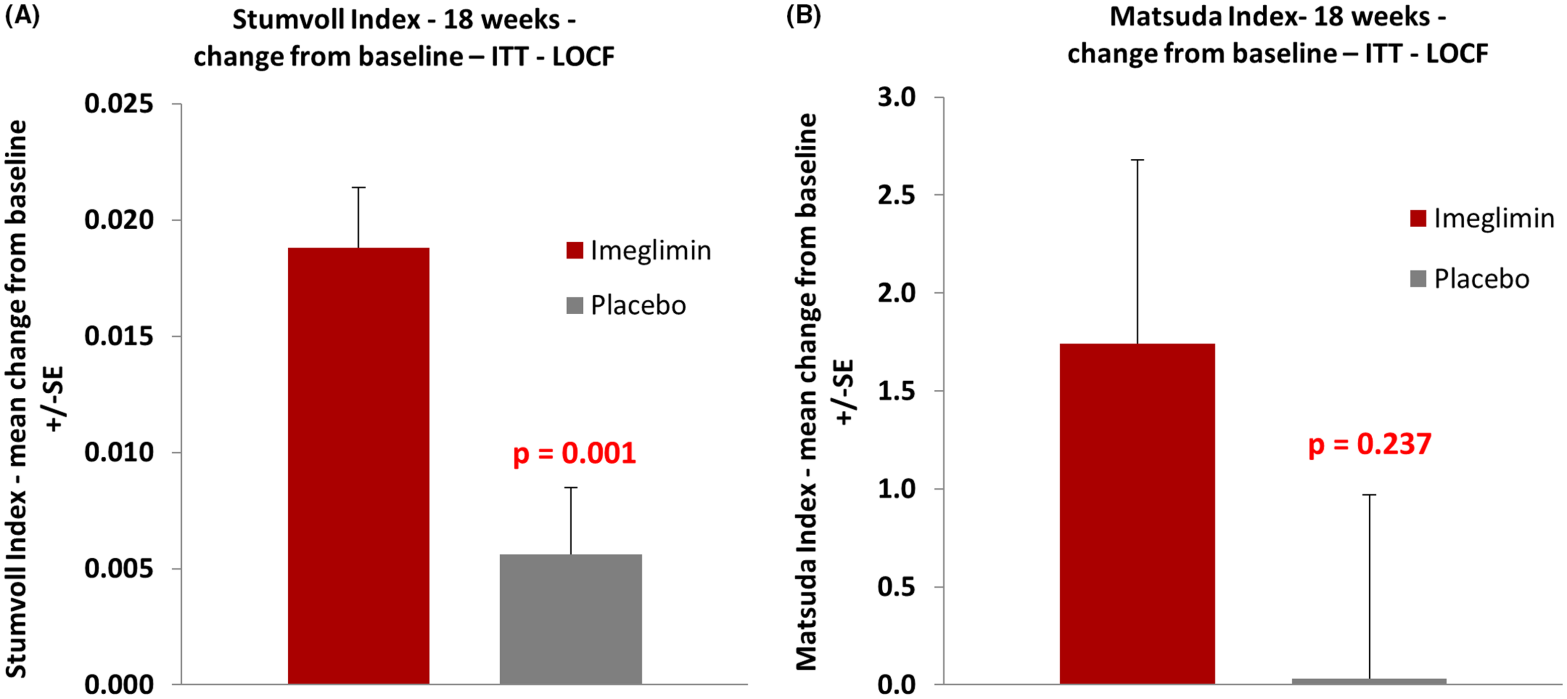
Effect of imeglimin on insulin sensitivity indices

To further investigate the mode of action of imeglimin on glucose homeostasis, we calculated several indices of insulin sensitivity. The QUICKI index for fasting insulin sensitivity showed numerical improvement but did not reach statistical significance (*p* = .244, Table 2). LS mean change from baseline to week 18 for the Stumvoll index was 0.0182 and 0.0049 in the imeglimin and placebo groups, respectively. LS mean difference between treatment groups was 0.0133 (95% CI: 0.0058, 0.0208), which was statistically significant (*p* = .001, Figure 6A, Table 2). For the Matsuda index, LS mean change from baseline to week 18 was 1.54 and − 0.02 in the imeglimin and placebo groups, respectively. LS mean difference between treatment groups was 1.55 (95% CI: −1.06, 4.17), which was not statistically significant due to high variability (*p* = .237, Figure 6B, Table 2). The OGIS index showed a more modest numerical improvement that was not significant (Table 2).

### 3.7 Efficacy at week 6

An optional visit was performed at week 6 to assess the primary and secondary efficacy parameters described above: OGTT, glycaemic parameters, insulin secretion and sensitivity. Even though no formal statistical comparison was performed at this time point, numerical results showed that at least 50% of the effect observed at week 18 ‐ on all the significantly altered parameters ‐ was achieved at week 6 for the imeglimin group (mean change from baseline –Table S1).

### 3.8 Safety

No life‐threatening events or deaths occurred during the study. The proportion of subjects experiencing a TEAE was higher in the placebo group compared with the imeglimin group: 8 (26.7%) subjects versus 17 (58.6%) subjects in the imeglimin and placebo groups respectively (Table S2). All reported TEAEs were mild to moderate in severity except 1 (3.4%) subject in the placebo group who experienced 1 TEAE (hyperglycaemia) of severe intensity. The most frequently reported TEAEs was hyperglycaemia in both treatment groups (5 [16.7%] subjects in the imeglimin group and 12 [41.4%] subjects in the placebo group). Most of the TEAEs were not treatment‐related, and all treatment‐related TEAEs were events of hyperglycaemia and were reported by 1 (3.3%) subject in the imeglimin group and 4 (13.8%) subjects in the placebo group (Table S3).

A higher percentage of subjects in the placebo group discontinued from the study due to therapeutic failure (the use of rescue medication for hyperglycaemic events) compared with the imeglimin group (1 (3.3%) subject in the imeglimin group and 10 (34.5%) subjects in the placebo group). No episode of hypoglycaemia was reported. One (3.3%) subject in the imeglimin group experienced 3 SAEs (cerebrovascular accident, hypertension and Meigs' syndrome), all considered unrelated to the study treatment; no subject in the placebo group experienced an SAE.

## 4 DISCUSSION

We endeavoured to further characterize imeglimin's efficacy profile and mode of action in a Phase 2 study where dynamic OGTT testing was performed and analysed. In line with previous preclinical and clinical evidence,^18^, ^31^ we established that an 18 weeks treatment period with imeglimin (1500 mg bid) in T2D patients improves glucose tolerance during a 3 h OGTT ‐ as shown by the marked decrease in glucose AUC. Thus, the primary endpoint of the study was achieved. Net placebo‐adjusted benefits on fasting glucose and HbA1c were also achieved in this trial. The effect on HbA1c (−0.62 vs. placebo) was relatively modest compared to other clinical studies in Japan. For example, in the 24 weeks phase 2b trial conducted in Japan, mean decreases in HbA1c vs. placebo were −0.94 and −1.0 with 1000 mg bid and 1500 bid, respectively ‐ and baseline HbA1c values were similar (7.7–7.9).^14^ This difference may be related to a shorter washout of metformin therapy ‐ only 4 weeks long, lesser compliance to diet and to drug, or different diabetes background and ethnic differences between Caucasian and Japanese subjects (i.e. a more prominent early defect in insulin secretion in Japanese subjects).

Although mean total insulin and C‐peptide AUC values during OGTT testing were not significantly affected (*p* = .052 for C‐peptide), clear beneficial effects involving stimulated insulin secretion in response to glucose, i.e. β‐cell function, were evident ‐ as shown by the increases in the AUC_0‐180min_ C‐peptide/AUC_0‐180min_ glucose, insulinogenic index and the model‐derived insulin secretion at 10 mmol/L glucose, rate sensitivity and glucose sensitivity. These effects of imeglimin confirm a clinical effect on the β‐cell function that we previously reported based on the use of hyperglycaemic clamp testing performed within a similar treatment time frame.^13^

Imeglimin improved the Stumvoll index of insulin sensitivity to a significant extent, while significance was not reached by the other surrogate indices. However, all indices concordantly showed a numerical improvement, suggesting that imeglimin likely also enhances insulin sensitivity. The fasting index of insulin sensitivity (QUICKI) was not significantly affected by imeglimin at variance with what was observed in the TIMES 1 pivotal monotherapy trial conducted in Japanese subjects^15^ where QUICKI improved. Since QUICKI is regarded as a measurement of liver insulin sensitivity,^32^ further studies are necessary to elucidate the role of the liver. From this study, for instance, no change in hepatic insulin extraction has been observed. It is also notable that ‐ although not statistically significant ‐ the mean change from baseline in Matsuda vs. placebo (+1.55) is similar in magnitude to that reported in multiple trials with pioglitazone, an established insulin sensitizer.^33^ These results fit nicely with observations during hyperinsulinemic clamp in rats,^18^ and await further confirmation with a clamp study in T2D subjects.

T2D aetiology derives from two primary factors: (1) the development of insulin resistance, and (2) impairment of glucose‐mediated insulin secretion by β‐cells in the pancreas.^1^ Commonly prescribed oral drugs acting on each of these components individually include thiazolidinediones (TZDs),^34^ and sulphonylurea or glinide insulin secretagogues.^35^ However, significant side effects including weight gain, oedema, heart failure and bone fracture risk limit the use of TZDs,^36^ while risks of hypoglycaemia and limited durability are well described for secretagogues. DPP4 inhibitors are well tolerated and act indirectly to enhance GSIS without hypoglycaemia risk; however, they are limited by more modest efficacy and shorter durability.^37^, ^38^ Metformin acts principally to reduce hepatic glucose overproduction ‐ an insulin‐like effect. Although it is widely used, metformin can be associated with GI tolerability issues and residual risk of lactic acidosis, especially in the context of impaired renal function.^5^ SGLT2 inhibitors are a recent and valuable class due to demonstrated cardio‐renal benefits; however, these agents have no specific‐direct effects on either β‐cell function or insulin action.^39^ In contrast, a substantial body of data supports the dual effects of imeglimin ‐ to both reverse β‐cell dysfunction (augmenting GSIS) and to enhance insulin action (in both liver and muscle).^18^ Taken together with previously published data, results from the present study provide clinical insight to support this dual action of imeglimin. This profile may be particularly appealing as a therapeutic option for T2D since its use will simultaneously target both key components of disease pathophysiology. Imeglimin may also be more broadly useful by encompassing potential inter‐individual and inter‐ethnic differences in disease aetiology, such as the prominent impairment of insulin secretion with an overall lesser degree of insulin resistance in East Asians compared to Caucasians.^40^, ^41^

No unexpected safety concerns were raised in this study and imeglimin was well tolerated. Imeglimin treatment showed a more favourable profile than placebo with regard to the incidence of TEAEs and the incidence of discontinuations due to TEAEs. Almost all TEAEs were mild or moderate in severity and the majority of TEAEs were not treatment‐related. Hyperglycaemia was the only event considered treatment‐related and occurred in only one subject in the imeglimin group. In addition, no events of hypoglycaemia were reported, in line with previous clinical results showing a low risk of hypoglycaemia.^15^, ^22^, ^23^ This observation is in accordance with preclinical results highlighting the effect of imeglimin on GSIS, and with the present results of this study providing evidence of increased early/first phase insulin release and β‐cell glucose sensitivity following an oral glucose load, with no effect on fasting insulin levels.

In conclusion, 1500 mg bid imeglimin treatment for 18 weeks significantly improves glucose tolerance; this is due to a substantial beneficial effect on insulin secretion in response to glucose and is also potentially contributed to by an impact on insulin sensitivity in T2D patients. A favourable safety profile was also observed. The potential dual mode of action of imeglimin on insulin secretion and sensitivity reported in this trial and in previously published preclinical and clinical studies support the attractiveness of imeglimin as a novel and innovative approach to improve the health of patients.

## AUTHOR CONTRIBUTIONS

**Pierre Theurey**: writing—original draft (lead), formal analysis (supporting), writing—review and editing (lead), validation (supporting). **Carole Thang**: formal analysis (lead), writing—review and editing (lead), validation (supporting). **Valdis Pirags**: conceptualization (supporting), investigation (lead), validation (supporting). **Andrea Mari**: conceptualization (lead), formal analysis (lead), writing—review and editing (supporting), validation (lead). **Giovanni Pacini**: conceptualization (lead), formal analysis (lead), writing—review and editing (supporting), validation (lead). **Sébastien Bolze**: conceptualization (supporting), formal analysis (lead), writing—review and editing (supporting), validation (supporting). **Sophie Hallakou‐Bozec**: conceptualization (supporting), formal analysis (supporting), writing—review and editing (lead), validation (supporting). **Pascale Fouqueray**: conceptualization (lead), formal analysis (lead), writing—review and editing (lead), validation (lead).

## FUNDING INFORMATION

The study presented was funded by Poxel.

## CONFLICT OF INTEREST

Pierre Theurey, Carole Thang, Sébastien Bolze, Sophie Hallakou‐Bozec and Pascale Fouqueray are employees and shareholders of Poxel. Valdis Pirags, Andrea Mari and Giovanni Pacini received fees from Poxel.

## Supporting information


Appendix S1

## Data Availability

The data that support the findings of this study are available from the corresponding author upon reasonable request.
