# Supplementary material for: Phase 2 trial with imeglimin in patients with Type 2 diabetes indicates effects on insulin secretion and sensitivity
Source: Endocrinol Diabetes Metab. 2022 Oct 14;5(6):e371. doi: 10.1002/edm2.371 (PMC9659655; doi:10.1002/edm2.371)
Supplement: Supplementary file 1 — Appendix S1 [file EDM2-5-e371-s001.docx]

**Supplementary Material**

**Endocrinology, Diabetes & Metabolism**

**EDM2-2022-0080**

**Phase 2 trial with Imeglimin in Patients with Type 2 Diabetes Indicates**

**Effects on Insulin Secretion and Sensitivity**

Endocrinology, Diabetes & Metabolism

Pierre Theurey^1*^, Carole Thang^1*^, Valdis Pirags^2^, Andrea Mari^3^, Giovanni Pacini^4^, Sébastien Bolze^1^, Sophie Hallakou-Bozec^1^, Pascale Fouqueray^1^

^1^ Poxel SA, Lyon, France

^2^ University of Latvia, Riga, Latvia

^3^ Institute of Neuroscience, National Research Council, Padova, Italy

^4^ Independent Researcher, Padova, Italy

**Supplemental Table 1. Effects of imeglimin versus placebo on primary and secondary efficacy end points at Week 6**

|  |  | **Imeglimin** | **Placebo** |
| --- | --- | --- | --- |
| 180-min AUC Glucose (mmol/L·min ), *n* | | 26 | 25 |
|  | Mean (SD) | 2609.3 (406.7) | 2926.8 (577.6) |
|  | Change from baseline, mean (SD) | -564.8 (468.6) | -166.6 (422.5) |
| 180-min iAUC Glucose (mmol/L·min ), *n* | | 26 | 25 |
|  | Mean (SD) | 1007.1 (390.9) | 1179.6 (382.1) |
|  | Change from baseline, mean (SD) | -281.0 (361.7) | -183.7 (310.1) |
| HbA1c (%), *n* | | 29 | 28 |
|  | Mean (SD) | 7.88 (0.69) | 8.32 (0.85) |
|  | Change from baseline, mean (SD) | -0.24 (0.43) | 0.18 (0.57) |
| Pre-dose Fasting Plasma Glucose (mmol/L), *n* | | 29 | 28 |
|  | Mean (SD) | 9.92 (1.71) | 10.23 (2.56) |
|  | Change from baseline, mean (SD) | -1.41 (2.18) | -0.01 (1.48) |
| Post-dose Fasting Plasma Glucose (mmol/L), *n* | | 27 | 24 |
|  | Mean (SD) | 9.31 (2.57) | 9.72 (2.35) |
|  | Change from baseline, mean (SD) | -1.36 (2.79) | 0.03 (1.52) |
| Pre-dose Fasting Insulin (pmol/L), *n* | | 29 | 26 |
|  | Mean (SD) | 10.67 (5.05) | 16.72 (14.76) |
|  | Change from baseline, mean (SD) | -1.16 (4.68) | -3.08 (11.83) |
| 180-min AUC Insulin (pmol/L·min ), *n* | | 25 | 23 |
|  | Mean (SD) | 7031.9 (4146.5) | 6201.4 (3174.8) |
|  | Change from baseline, mean (SD) | 1589.3 (3065.5) | 5.0 (1428.4) |
| 180-min AUC C-peptide (nmol/L·min ), *n* | | 25 | 24 |
|  | Mean (SD) | 420.0 (133.7) | 346.1 (117.0) |
|  | Change from baseline, mean (SD) | 45.0 (86.1) | -16.1 (61.0) |
| Stumvoll index, *n* | | 25 | 24 |
|  | Mean (SD) | 0.0578 (0.0166) | 0.0461 (0.0208) |
|  | Change from baseline, mean (SD) | 0.0132 (0.0101) | 0.0039 (0.0122) |
| Matsuda index, *n* | | 24 | 21 |
|  | Mean (SD) | 2.35 (1.47) | 2.03 (1.34) |
|  | Change from baseline, mean (SD) | -0.39 (0.95) | 0.17 (1.02) |
| OGIS index (2-h equation – mL/min/m²), *n* | | 24 | 21 |
|  | Mean (SD) | 267.86 (48.40) | 281.49 (47.26) |
|  | Change from baseline, mean (SD) | 4.05 (47.53) | 4.58 (36.57) |
| QUICKI, *n* | | 29 | 26 |
|  | Mean (SD) | 0.225 (0.031) | 0.209 (0.028) |
|  | Change from baseline, mean (SD) | 0.007 (0.031) | 0.005 (0.023) |
| Insulinogenic index (pmol/mmol), *n* | | 24 | 21 |
|  | Mean (SD) | 3.86 (4.41) | 2.49 (2.59) |
|  | Change from baseline, mean (SD) | 1.55 (4.48) | 0.44 (1.51) |
| AUC0-180min C-peptide / AUC0-180min glucose (nmol/mmol), *n* | | 25 | 24 |
|  | Mean (SD) | 0.163 (0.054) | 0.121 (0.047) |
|  | Change from baseline, mean (SD) | 0.041 (0.039) | 0.002 (0.020) |
| Rate sensitivity (pmol/m²/mmol/L), *n* | | 25 | 24 |
|  | Mean (SD) | 313.58 (326.21) | 247.00 (205.22) |
|  | Change from baseline, mean (SD) | 93.52 (311.73) | 34.85 (227.12) |
| Insulin Secretion at 10 mmol/L Glucose from the Dose-Response (pmol/min/m²), *n* | | 25 | 24 |
|  | Mean (SD) | 162.09 (76.58) | 142.18 (75.16) |
|  | Change from baseline, mean (SD) | 30.57 (52.35) | -5.72 (46.28) |
| Glucose sensitivity (pmol/min/m²/mmol/L), *n* | | 25 | 24 |
|  | Mean (SD) | 35.34 (16.51) | 20.13 (10.40) |
|  | Change from baseline, mean (SD) | 14.29 (17.53) | 1.09 (8.32) |

AUC: area under the curve; HbA1c=glycated haemoglobin; iAUC=incremental area under the curve; SD=standard deviation

| **Supplemental Table 2. Adverse events overview** | | | | | | | | |
| --- | --- | --- | --- | --- | --- | --- | --- | --- |
|  | Imeglimin (N=30) | |  | Placebo (N=29) | |  | Total (N=59) | |
|  | Subjects (%) | Events |  | Subjects (%) | Events |  | Subjects (%) | Events |
|  |  |  |  |  |  |  |  |  |
| Any AEs | 13 (43.3) | 53 |  | 19 (65.5) | 40 |  | 32 (54.2) | 93 |
| Any TEAEs | 8 (26.7) | 24 |  | 17 (58.6) | 25 |  | 25 (42.4) | 49 |
| Any TEAEs Leading to Study Discontinuation | 1 (3.3) | 1 |  | 10 (34.5) | 10 |  | 11 (18.6)^1^ | 11 |
| Any Related TEAEs | 1 (3.3) | 1 |  | 4 (13.8) | 5 |  | 5 (8.5) | 6 |
| Any SAEs | 1 (3.3) | 3 |  | 0 | 0 |  | 1 (1.7) | 3 |
| Any Severe AEs | 0 | 0 |  | 1 (3.4) | 1 |  | 1 (1.7) | 1 |
| Any Cardiovascular AEs | 1 (3.3) | 1 |  | 0 | 0 |  | 1 (1.7) | 1 |
|  |  |  |  |  |  |  |  |  |

AE=adverse event; SAE=serious adverse event; TEAE=treatment-emergent adverse event.

**Supplemental Table 3. Summary of treatment emergent adverse events by System Organ Class and Preferred Term**

| SOC/  PT | Imeglimin (N=30) | | Placebo (N=29) | | Total (N=59) | |
| --- | --- | --- | --- | --- | --- | --- |
|  | Subjects (%) | Events | Subjects (%) | Events | Subjects (%) | Events |
|  |  |  |  |  |  |  |
| Subjects with Any TEAEs | 8 (26.7) | 24 | 17 (58.6) | 25 | 25 (42.4) | 49 |
|  |  |  |  |  |  |  |
| **Cardiac disorders** | 3 (10.0) | 9 | 0 | 0 | 3 (5.1) | 9 |
| Aortic valve sclerosis | 1 (3.3) | 1 | 0 | 0 | 1 (1.7) | 1 |
| Cardiac failure chronic | 1 (3.3) | 1 | 0 | 0 | 1 (1.7) | 1 |
| Diastolic dysfunction | 1 (3.3) | 1 | 0 | 0 | 1 (1.7) | 1 |
| Hypertensive heart disease | 1 (3.3) | 1 | 0 | 0 | 1 (1.7) | 1 |
| Left ventricular hypertrophy | 2 (6.7) | 2 | 0 | 0 | 2 (3.4) | 2 |
| Mitral valve incompetence | 1 (3.3) | 1 | 0 | 0 | 1 (1.7) | 1 |
| Mitral valve sclerosis | 1 (3.3) | 1 | 0 | 0 | 1 (1.7) | 1 |
| Tricuspid valve incompetence | 1 (3.3) | 1 | 0 | 0 | 1 (1.7) | 1 |
|  |  |  |  |  |  |  |
| **Endocrine disorders** | 1 (3.3) | 1 | 0 | 0 | 1 (1.7) | 1 |
| Goitre | 1 (3.3) | 1 | 0 | 0 | 1 (1.7) | 1 |
|  |  |  |  |  |  |  |
| **Infections and infestations** | 2 (6.7) | 2 | 4 (13.8) | 4 | 6 (10.2) | 6 |
| Bronchitis | 0 | 0 | 1 (3.4) | 1 | 1 (1.7) | 1 |
| Escherichia urinary tract infection | 0 | 0 | 1 (3.4) | 1 | 1 (1.7) | 1 |
| Nasopharyngitis | 0 | 0 | 1 (3.4) | 1 | 1 (1.7) | 1 |
| Respiratory tract infection | 1 (3.3) | 1 | 0 | 0 | 1 (1.7) | 1 |
| Urinary tract infection | 1 (3.3) | 1 | 1 (3.4) | 1 | 2 (3.4) | 2 |
|  |  |  |  |  |  |  |
| **Investigations** | 0 | 0 | 1 (3.4) | 1 | 1 (1.7) | 1 |
| Electrocardiogram QT prolonged | 0 | 0 | 1 (3.4) | 1 | 1 (1.7) | 1 |
|  |  |  |  |  |  |  |
| **Metabolism and nutrition disorders** | 5 (16.7) | 7 | 13 (44.8) | 14 | 18 (30.5) | 21 |
| Hypercholesterolaemia | 0 | 0 | 1 (3.4) | 1 | 1 (1.7) | 1 |
| Hyperglycaemia | 5 (16.7) | 7 | 12 (41.4) | 13 | 17 (28.8) | 20 |
|  |  |  |  |  |  |  |
| **Musculoskeletal and connective tissue disorders** | 0 | 0 | 1 (3.4) | 1 | 1 (1.7) | 1 |
| Spinal osteoarthritis | 0 | 0 | 1 (3.4) | 1 | 1 (1.7) | 1 |
|  |  |  |  |  |  |  |
| **Neoplasms benign, malignant and unspecified (incl cysts and polyps)** | 1 (3.3) | 1 | 0 | 0 | 1 (1.7) | 1 |
| Meigs’ syndrome | 1 (3.3) | 1 | 0 | 0 | 1 (1.7) | 1 |
|  |  |  |  |  |  |  |
| **Nervous system disorders** | 1 (3.3) | 2 | 3 (10.3) | 3 | 4 (6.8) | 5 |
| Cerebral ischaemia | 1 (3.3) | 1 | 0 | 0 | 1 (1.7) | 1 |
| Cerebrovascular accident | 1 (3.3) | 1 | 0 | 0 | 1 (1.7) | 1 |
| Diabetic neuropathy | 0 | 0 | 3 (10.3) | 3 | 3 (5.1) | 3 |
|  |  |  |  |  |  |  |
| **Psychiatric disorders** | 0 | 0 | 1 (3.4) | 1 | 1 (1.7) | 1 |
| Anxiety disorder due to a general medical condition | 0 | 0 | 1 (3.4) | 1 | 1 (1.7) | 1 |
|  |  |  |  |  |  |  |
| **Vascular disorders** | 1 (3.3) | 2 | 1 (3.4) | 1 | 2 (3.4) | 3 |
| Aortic arteriosclerosis | 1 (3.3) | 1 | 0 | 0 | 1 (1.7) | 1 |
| Hypertension | 1 (3.3) | 1 | 1 (3.4) | 1 | 2 (3.4) | 2 |

AE=adverse event; PT=Preferred Term; SOC=System Organ Class; TEAE=treatment‑emergent adverse event.

**
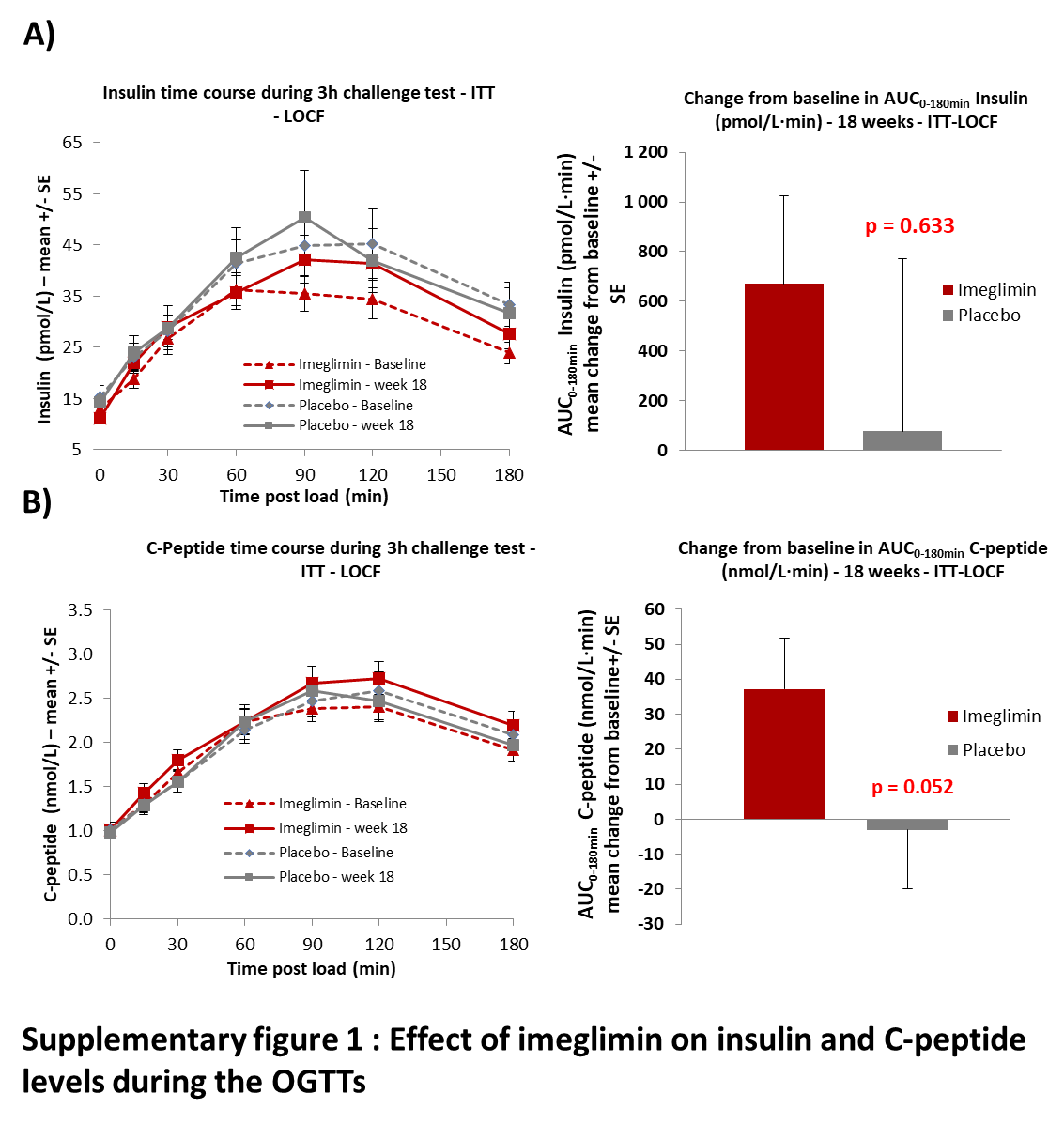
**
